# Anorectal Malformations (Part 2)

**Published:** 2015-04-01

**Authors:** Sushmita Bhatnagar

**Affiliations:** Department of Pediatric Surgery, B.J.Wadia Hospital for Children, Mumbai

 (This section is meant for residents to check their understanding regarding a particular topic)

## Questions

Q.1. What are the pre-operative workup/investigations necessary for a baby born with ARM?
Q. 2. Briefly describe the aims and details of surgical management of anorectal malformations.

## Answers

**Answer 1**

As discussed in the first part, anorectal malformations represent a wide spectrum of anomalies involving more than one system. Also, the pre-operative work up differs in boys and girls (Tables 1 and 2). The pre-operative work up could be discussed under the following headings:

a) For diagnosis of type of anorectal anomaly
b) For associated anomalies
c) For spinal abnormalities
d) For perineal musculature

**Figure:**
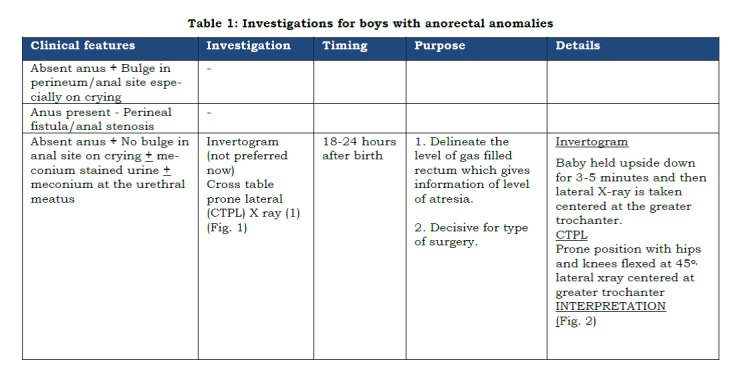
Table 1: Investigations for boys with anorectal anomalies.

**Figure:**
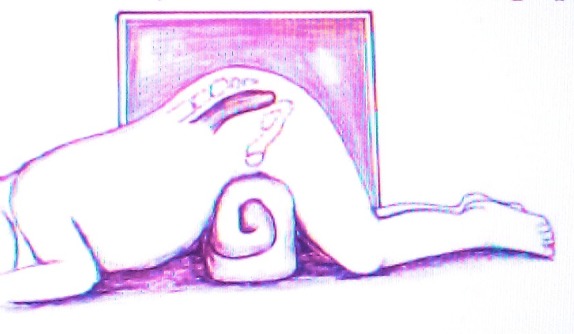
Figure 1: Positioning of the baby for Cross Table Prone Lateral X-Ray.

**Figure:**
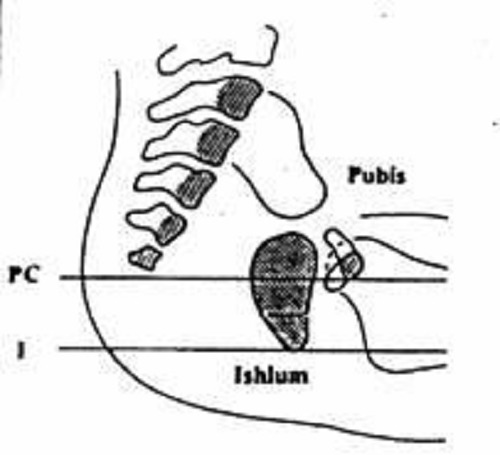
Figure 2: Interpretation of Invert gram or CTPL X-ray.

**Figure:**
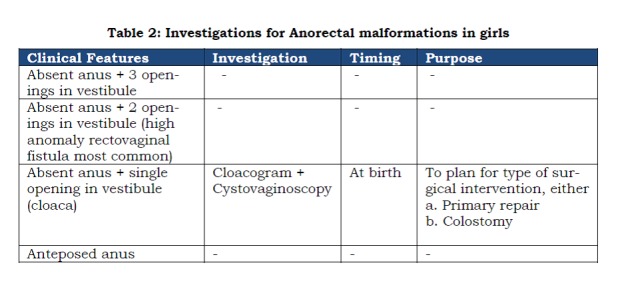
Table 2: Investigations for Anorectal malformations in girls

Some of the associated anomalies significantly impact the overall outcome in patients with anorectal malformations, so they must be evaluated at birth (Table 3). 

**Figure:**
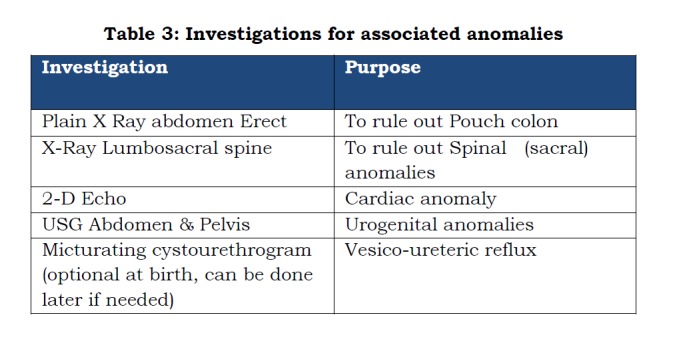
Table 3: Investigations for associated anomalies

Preoperative MRI of the pelvis and perineum is indicated for perineal musculature and the delineation of the sphincter complex in selected cases. MRI is helpful in thorough evaluation of the following (2):

a. Quality and shape of muscles responsible for fecal continence.
b. Location of bowel and its relation to the muscle complex.
c. Level of fistula and posterior urethral diverticulum.
d. Sacral spinal anomalies, if any.
e. Associated genitourinary anomalies.

Apart from pre-operative evaluation, MRI also assists in prognostication of the long-term outcome and the quality of life of the child with anorectal malformation.

**Answer 2 **

The most important aim of the surgical correction is to create a normal anus with anatomic reconstruction. Surgery should help the child to achieve a socially acceptable bowel function and should ensure avoiding fecal incontinence, urinary incontinence or sexual dysfunction.

The choices of surgical correction are as follows:

a. Primary repair – both boys and girls (3-6)
b. Staged repair – usually 3 stages:
  i. Colostomy – most probably high sigmoid loop in left iliac fossa.
ii. Pull through – Posterior sagittal approach, abdomino-perineal approach, abdominal posterior sagittal approach, laparoscopic approach – in boys; anterior sagittal approach, anal transposition, posterior sagittal, abdomino-perineal, abdominal posterior sagittal approach – in girls.
iii. Colostomy closure.

The various surgical techniques that have been used for the management of anorectal malformations have been tabulated below:

**Figure:**
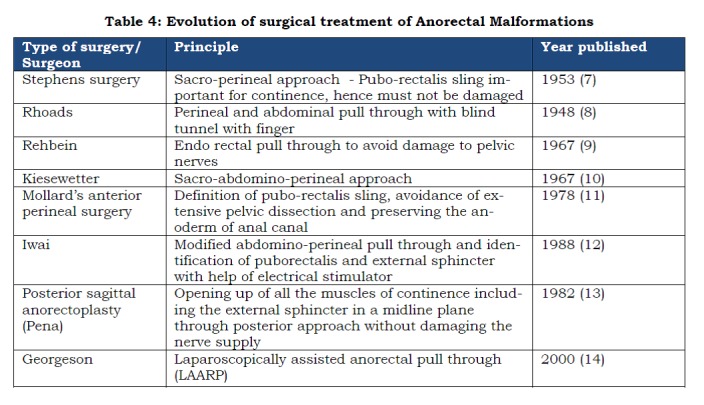
Table 4: Evolution of surgical treatment of Anorectal Malformations

To summarize, the management of anorectal malformations, which differs in boys and girls, algorithms are presented for each respectively (Fig. 3 and 4)

**Figure:**
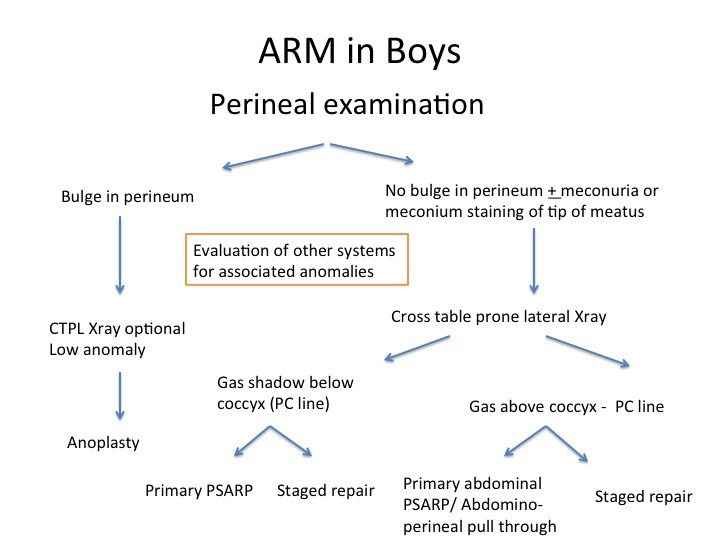
Figure 3: Algorithm for management of Male ARM at birth.

**Figure:**
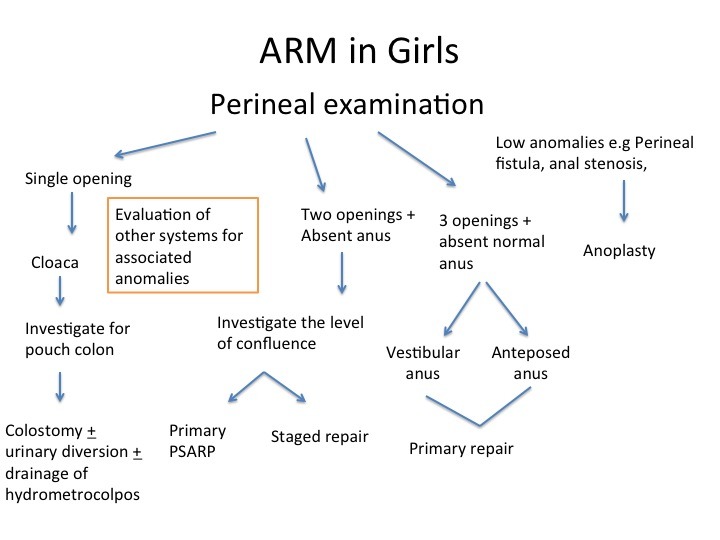
Figure 4: Algorithm for management of female Anorectal malformations at birth.

## Footnotes

**Source of Support:** Nil

**Conflict of Interest:** The author is editor of the journal. The manuscript is independently handled by other editors and she is not involved in decision making about the manuscript.

